# Bisphosphonates in the Adjuvant Setting of Breast Cancer Therapy—Effect on Survival: A Systematic Review and Meta-Analysis

**DOI:** 10.1371/journal.pone.0070044

**Published:** 2013-08-26

**Authors:** Irit Ben-Aharon, Liat Vidal, Shulamith Rizel, Rinat Yerushalmi, Ofer Shpilberg, Aaron Sulkes, Salomon M. Stemmer

**Affiliations:** 1 Institute of Oncology, Davidoff Center, Rabin Medical Center, Petah-Tiqva, Israel; 2 Institute of Hematology, Davidoff Center, Rabin Medical Center, Petah-Tiqva, Israel; 3 Sackler Faculty of Medicine, Tel Aviv University, Tel Aviv, Israel; University Medical Center Utrecht, The Netherlands

## Abstract

**Background:**

The role of bisphosphonates (BP) in early breast cancer (BC) has been considered controversial. We performed a meta-analysis of all randomized controlled trials (RCTs) that appraised the effects of BP on survival in early BC.

**Methods:**

RCTs were identified by searching the Cochrane Library, MEDLINE databases and conference proceedings. Hazard ratios (HRs) of overall survival (OS), disease-free survival (DFS) and relative risks of adverse events were estimated and pooled.

**Results:**

Thirteen trials met the inclusion criteria, evaluating a total of 15,762 patients. Meta-analysis of ten trials which reported OS revealed no statistically significant benefit in OS for BP (HR 0.89, 95% CI = 0.79 to 1.01). Meta-analysis of nine trials which reported the DFS revealed no benefit in DFS (HR 0.95 (0.81–1.12)). Meta-analysis upon menopausal status showed a statistically significant better DFS in the BP-treated patients (HR 0.81(0.69–0.95)). In meta-regression, chemotherapy was negatively associated with HR of survival.

**Conclusions:**

Our meta-analysis indicates a positive effect for adjuvant BP on survival only in postmenopausal patients. Meta-regression demonstrated a negative association between chemotherapy use BP effect on survival. Further large scale RCTs are warranted to unravel the specific subgroups that would benefit from the addition of BP in the adjuvant setting.

## Introduction

Breast cancer is the most common malignancy among women, accounting for nearly 1 in 3 cancers diagnosed and the second leading cause of cancer death among women in the United States [1]. Survival rates are highly correlated with the extent of the disease at diagnosis whereas early stage disease confers superior survival rates. The majority of patients with advanced breast cancer eventually develop bone metastases. Breast cancer has a particular propensity for the bone; likewise, it has been previously demonstrated that the relationship between cancer cells and the bone microenvironment is mediated by a reciprocal interaction between cancer cells and normal bone cells [2]–[4]. Due to their beneficial effects on bone turnover, bisphosphonates have been evaluated for the prevention and treatment of bone metastases in women with breast cancer [5]–[8]. Two large population-based cohort studies demonstrated a significant reduction in the incidence of breast cancer in women who were treated with BP for non-cancer indications for more than a year [9]–[10]. Both pre-clinical and clinical evidence indicate that BP exhibit anti-metastatic and anti-tumor properties, including the inhibition of angiogenesis, and a unique effect in the bony niche – inhibition of cancer cell invasion and adhesion and the induction of apoptosis [11]–[12]. The role of BP in addition to standard adjuvant treatment has been evaluated in several studies, yielding inconsistent evidence regarding the antitumor effect of BP [13]–[29].

In view of the conflicting data from randomized clinical trials, we performed a systematic review of the literature and a meta-analysis of all randomized trials to evaluate the impact of BP administration on survival in patients with early breast cancer in the adjuvant setting.

## Methods

### Data Sources

We searched the Cochrane Central Register of Controlled Trials, published in The Cochrane Library, PubMed (1966 to 4.2012); EMBASE (1974 to 7.2012); LILACS (1982 to 7.2012); the database of clinical trials in non-metastatic breast cancer; conference proceedings of the American Society of Clinical Oncology (1995–7.2012), San Antonio Breast Cancer Symposium (1995–2012), proceedings of the European Society of Medical Oncology (ESMO); and databases of ongoing and unpublished trials: http://www.clinicaltrials.gov and ,http://www.clinicaltrials.nci.nih.gov. The terms (early OR adjuvant) AND (breast OR mammary) AND (tumor OR malign* OR carcinoma* OR cancer) AND (biphosphonates OR bisphosphonates OR clodronate OR pamidronate OR zoledronic acid OR ibandronate) were cross-searched. We scanned references of all included trials and reviews identified for additional studies.

### Study Selection

We included randomized controlled trials that compared BP adjuvant therapy in addition to the standard therapy (cytotoxic or hormonal) with placebo in patients with early breast cancer in the adjuvant setting. We included trials regardless of publication status, date of publication, and language. Two authors (IBA and LV) independently inspected each reference title identified by the search and applied the inclusion criteria.

### Data Extraction and Quality Assessment

Trials that fulfilled the inclusion criteria were assessed for methodological quality by two authors (IBA and LV). Information about randomization and allocation concealment, blinding, sample size, exclusions after randomization, and the length of follow-up were recorded [30]. The same two authors independently extracted the data from publications of included trials. The data extraction was discussed, decisions were documented, and, if necessary, the authors of the trials were contacted for clarification. Authors of included trials were contacted for all data relevant to the primary and secondary outcomes (survival and safety data) of this study and quality variables. In case of several publications for the same trial, the most updated one was extracted. Safety outcomes were pooled from the most updated publication of every trial.

### Outcome Measures

The primary outcome was overall survival, which was defined as time from randomization to death. Secondary outcomes were disease-free survival (defined as the time from randomization to earliest occurrence of relapse or death from any cause), and toxicity (defined as grade 3 or 4 hematological and nonhematological adverse events).

### Data Synthesis and Statistical Analysis

Hazard ratios (HRs) and variances for time-to-event outcomes were estimated as described by Parmar et al. [31] and pooled according to inverse of variance method (Review Manager [RevMan], version 5.1(Copenhagen: The Nordic Cochrane Centre, The Cochrane Collaboration, 2011). A HR less than 1.0 was in favor of bisphosphonate therapy. Relative risks (RRs) and 95% confidence intervals (CIs) for dichotomous data were estimated using the Mantel – Haenszel method [31]. We used a fixed effect model. In case of performing a sensitivity analysis for the primary outcomes we repeated the above analysis using a random effects model (the DerSimonian and Laird method) [32]. Since the Mantel-Haenszel odds ratio method using a 0.5 zero-cell correction were shown to be biased when events are rare, we therefore performed for rare events as osteonecrosis of the jaw (ONJ) a sensitivity analysis by using Peto method [33]. We assessed heterogeneity of trial results by the chi test of heterogeneity and the *I* ^2^ statistic of inconsistency. Statistically significant heterogeneity was defined as *P* less than .1 or an *I* ^2^ statistic greater than 50% [32]. In case of a significant heterogeneity (*I* ^2^>50%) we used a random-effects model. Potential sources of heterogeneity were explored through stratifying by type of induction therapy (use of chemotherapy and endocrine therapy, endocrine therapy only, menopausal status), bisphosphonate's schedule and type of BP, allocation concealment, blinding, and size of studies. All statistical tests were two-sided.

We explored potential ground of heterogeneity through meta-regression, assessing the effect of percentage of patients receiving chemotherapy in addition to endocrine therapy in each study on the effect estimates for the primary outcome. Meta-regression was performed on the HR (Comprehensive Meta Analysis, version 2.2; BioStat, Englewood, NJ). The regression slope with its SE and significance of the model are reported.

## Results

**Figure 1:**
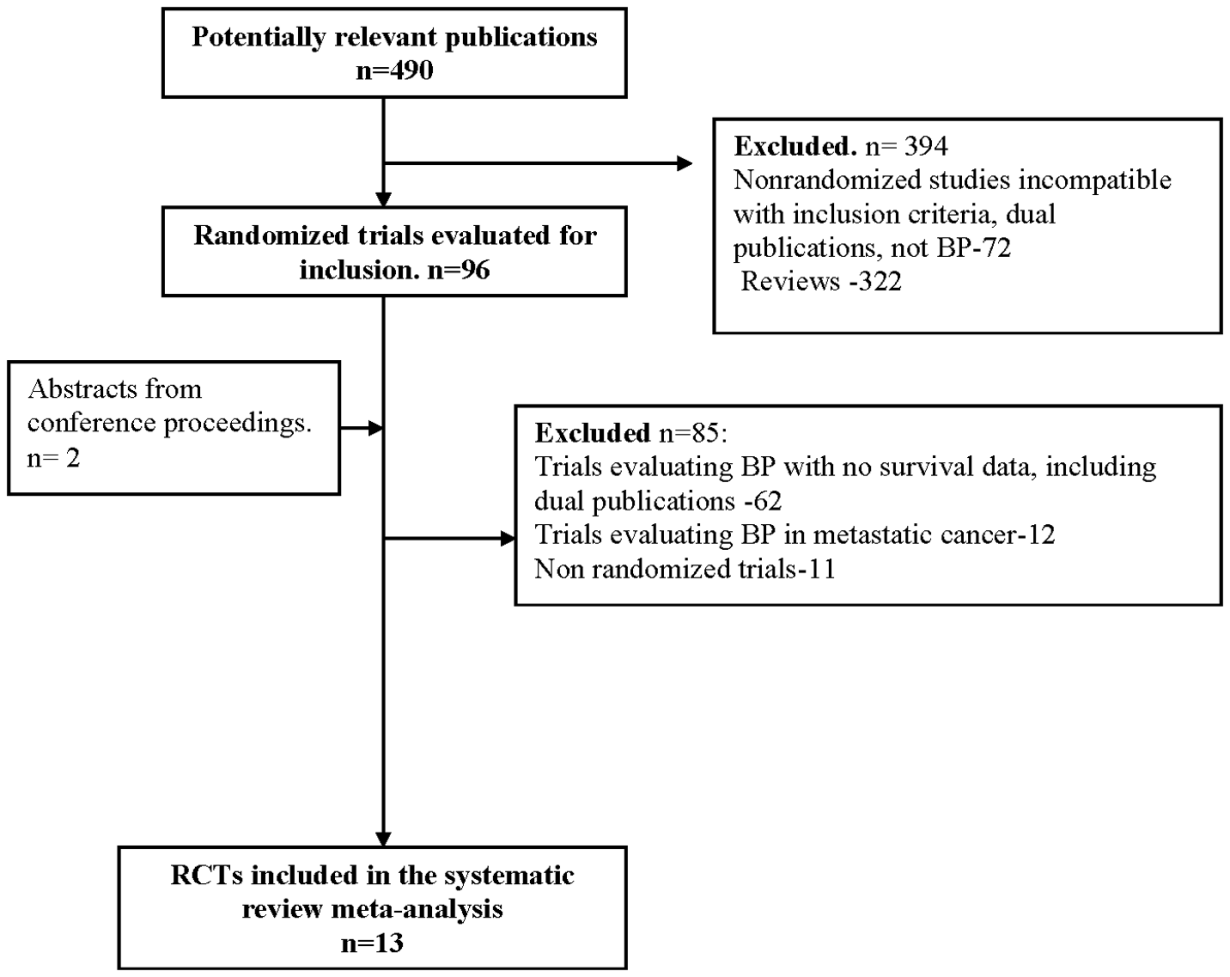
Randomized controlled trials search and selection.

The literature search identified 490 trials up until 7.2012, of which 96 were considered potentially relevant. Additional trials were identified by searching conference proceedings and electronic resources of ongoing trials. Figure 1 illustrates the process of study selection. Thirteen trials were designed to evaluate the effect of bisphosphonates on survival and fulfilled the inclusion criteria for published studies ([13]–[29]; including safety reports) including two recently published abstracts of large scale randomized controlled trials [28]–[29].

### Studies' characteristics

A total of 15,762 patients were randomly assigned in the thirteen trials included in this meta-analysis. Nevertheless, in 3 studies disease free survival has been the primary endpoint with no report of overall survival data [18], [20]–[21], [26]–[27].

### Trial Design

In six trials [18]–[20], [22], [24], [26], patients were randomly assigned to zoledronic acid in addition to standard- of-care therapy. Four trials used clodronate, [14]–[15], [17], [28], one trial used pamidronate [16] and one risedronate [13] and one ibandronate [29]; the schedule varied as reflected in Table 1.

**Table 1:** Included studies' characteristics.

First Author, Year, Publication status	BP, Schedule	Duration of Treatment	Number of Patients BP/Placebo (or delayed)	Follow Up (median, moths)	Menopausal Status (% - post)	Adjuvant Chemotherapy (%, mean)	Adjuvant Endocrine Treatment
Delmas, 1997	PO Risedronate	2 y	27/26	36 m	100	100	Tamoxifen
Published PR	30 mg/d for 2 wk then 10 w off						
Saarto, 2004	PO Clodronate	3 y	139/143	120 m	47.7	54	Tamoxifen
Published PR	1600 mg/d						
Powels, 2006	PO Clodronate	2 y	530/539	66 m	50	74	NS
Published PR	1600 mg/d						
Diel,	PO Clodronate	2 y	157/145	103 m	62.5	42.1	Tamoxifen
2008	1600 mg/d						
Published PR							
Kristensen, 2008	PO Pamidronate	4 y	460/493	120 m	33	100	NS
Published PR	150bid						
Brufsky,	Upfront IV zolendronic acid vs. Delayed zolendronic acid	5 y	300/300	61 m	100	47	Letrozole
Z-FAST, 2011	4 mg q6m						
Published PR							
Coleman EZO-FAST, 2009	Upfront IV zolendronic acid vs. Delayed zolendronic acid	5 y	252/270	36 m	100	53	Letrozole
Conference proceeding	4 mg q6m						
Leal,	IV zolendronic acid	1 y	36/32	96 m	100	90	Tamoxifen
2010???	4 mg q3m						(AI – 15%)
Published PR							
Eidtmann, 2010,	Upfront IV zolendronic acid vs. Delayed zolendronic acid	5 y	532/533	60 m	100	54	Letrozole
ZO-FAST	4 mg q6m						
Published PR							
+ update from SABCS 2011							
Gnant, ABCSG12, 2011	IV zolendronic acid	3 y	899/904	84 m	0	5.5	Tamoxifen
Published PR	4 mg q6m						Anastrazole
+ update from SABCS 2011							GnRH* agonist
Coleman, AZURE, 2011	IV zolendronic acid	5 y	1680/1680	59 m	55	96	NS
Published PR	4 mg q6m						
NSABP B34, 2012	PO Clondronate	3 y	1662/1661	101 m	64	68	NS
	1600 mg/d						
GAIN,	PO Ibandronate	2 y	1996/998	39 m	52	100	NS
2011, Conference proceeding	50 mg/d						

PO – Per os (oral); IV - intravenous; PR – peer review; NS- not specified; GnRH – Gonadotropin Releasing Hormone AI – Aromatase inhibitor.

### Quality of Trials

Allocation concealment was reported as adequate in five trials [15], [22], [24], [28]–[29] and was not reported in the other eight trials. Few of the trials have been open-labeled. The quality assessment of the included trials has been performed according to the Cochrane risk of bias tool, as described in detail in Table S1. We appraised the rate of patients lost to follow up and in the majority of the studies the rate was <10%. In three trials the rate was higher (Range – 17–42%; [19]–[20], [28]).

### Overall Survival

**Figure 2:**
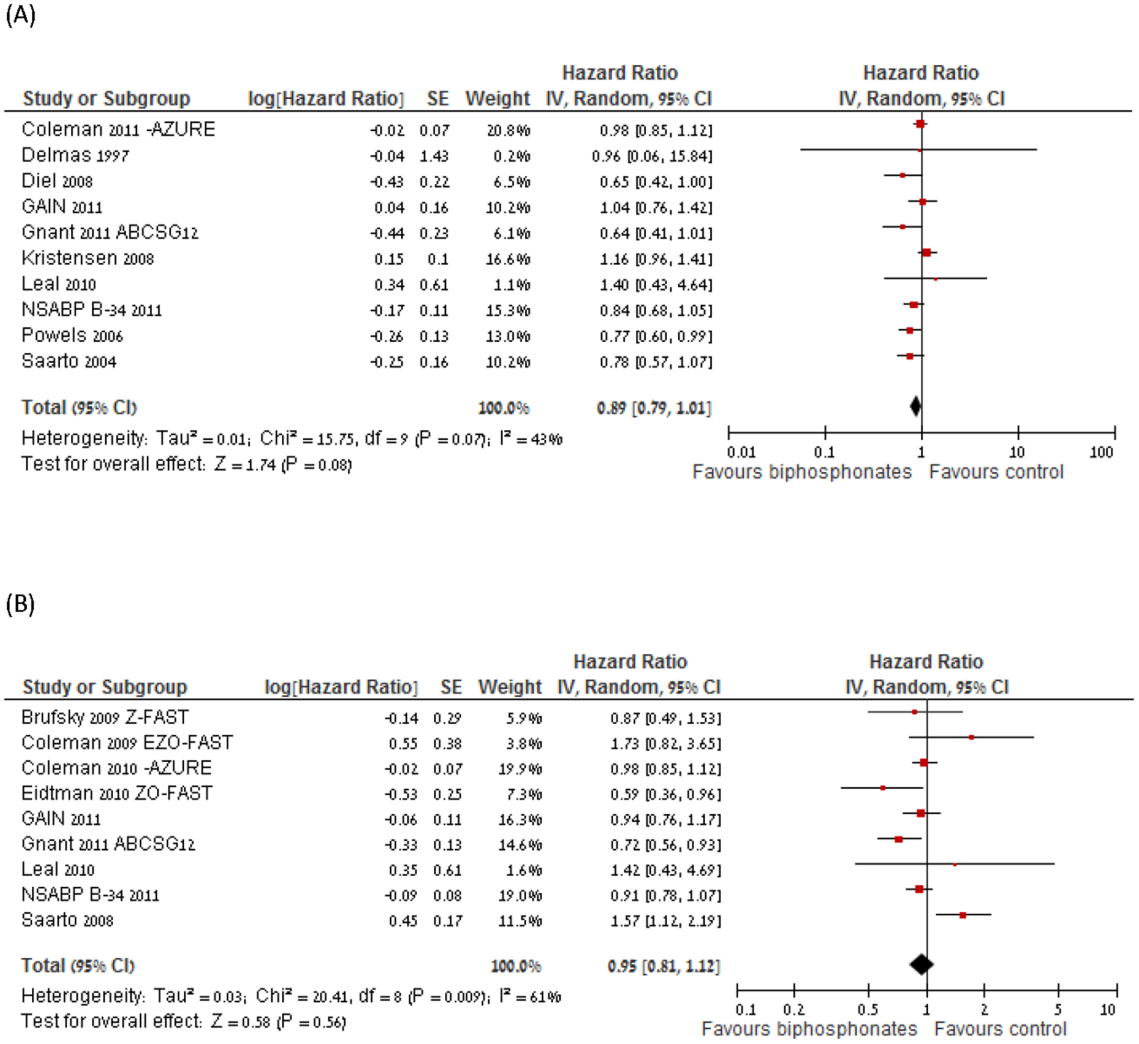
Forest plot of hazard ratios (HRs) comparing (A) overall survival or (B) disease-free survial (DFS) for patients who received BP in addition to standard therapy vs. those who received standard therapy only. Hazard ratios for each trail are represented by the **squares**, the size of the square represents the weight of the trial in the meta-analysis, and the **horizontal line** crossing the square represents the 95% confidence interval (CI). The **diamonds** represent the estimated overall effect based on the meta-analysis random effect of all trials.

Ten trials (13,571 patients) were eligible for the meta-analysis of overall survival [13]–[17], [19], [22], [24], [28]–[29]. The numbers of randomly assigned and analyzed patients in each included trial are described in Table 1. Patients who were treated with BP therapy had no statistically significant different overall survival than patients in the control group (HR of death = 0.89, 95% CI = 0.79 to 1.01; *I^2^* of heterogeneity −43%, random effect model) (Figure 2a). Sensitivity analysis of two trials that had short duration of follow up [13], [19] demonstrated that HR remained robust lacking these trials (HR of death = 0.89, 95% CI = 0.77 to 1.01, compared with 0.89, 0.79–1.01), presented in Figure S1). The funnel plot of the primary outcome did not support a publication bias.

### Disease Free Survival

Nine trials (12,167 patients) were eligible for the meta-analysis of DFS [14], [18]–[20], [22], [24], [26], [28]–[29]. The numbers of randomly assigned and analyzed patients in each included trial are described in Table 1. Patients who were treated with BPtherapy had no statistically significant better DFS than patients in the control group (HR of death = 0.95, 95% CI = 0.81 to 1.12; *I^2^* of heterogeneity −61%, random effect model) (Figure 2b). The funnel plot of the primary outcome did not support a publication bias.

### Subgroup analysis according to menopausal status

**Figure 3:**
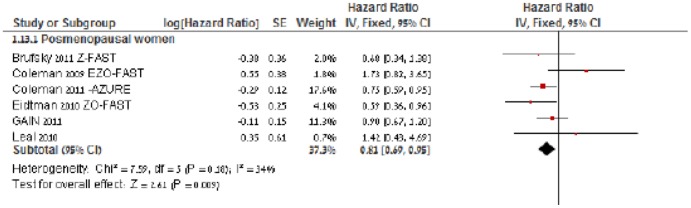
Forest plot of hazard ratios comparing disease-free survival for most-menopausal patients who received BP in addition to standard therapy vs. those who received standard therapy only. Hazard ratios for each trail are represented by the **squares**, the size of the square represents the weight of the trial in the meta-analysis, and the **horizontal line** crossing the square represents the 95% confidence interval (CI). The **diamonds** represent the estimated overall effect based on the meta-analysis random effect of all trials.

Six trials reported the DFS outcome stratified by menopausal status [18]–[20], [24], [26], [29]. Postmenopausal patients who were treated with BP therapy had statistically significant better DFS than patients in the control group (HR of death = 0.81, 95% CI = 0.69 to 0.95; *I^2^* of heterogeneity −34%, fixed effect model) (Figure 3). Meta-analysis for overall survival stratified by postmenopausal status was feasible for two trials only, and indicated a favorable outcome for the intervention arm (HR of death = 0.74, 95% CI = 0.57 to 0.98).

### Interaction with potential confounders

**Figure 4:**
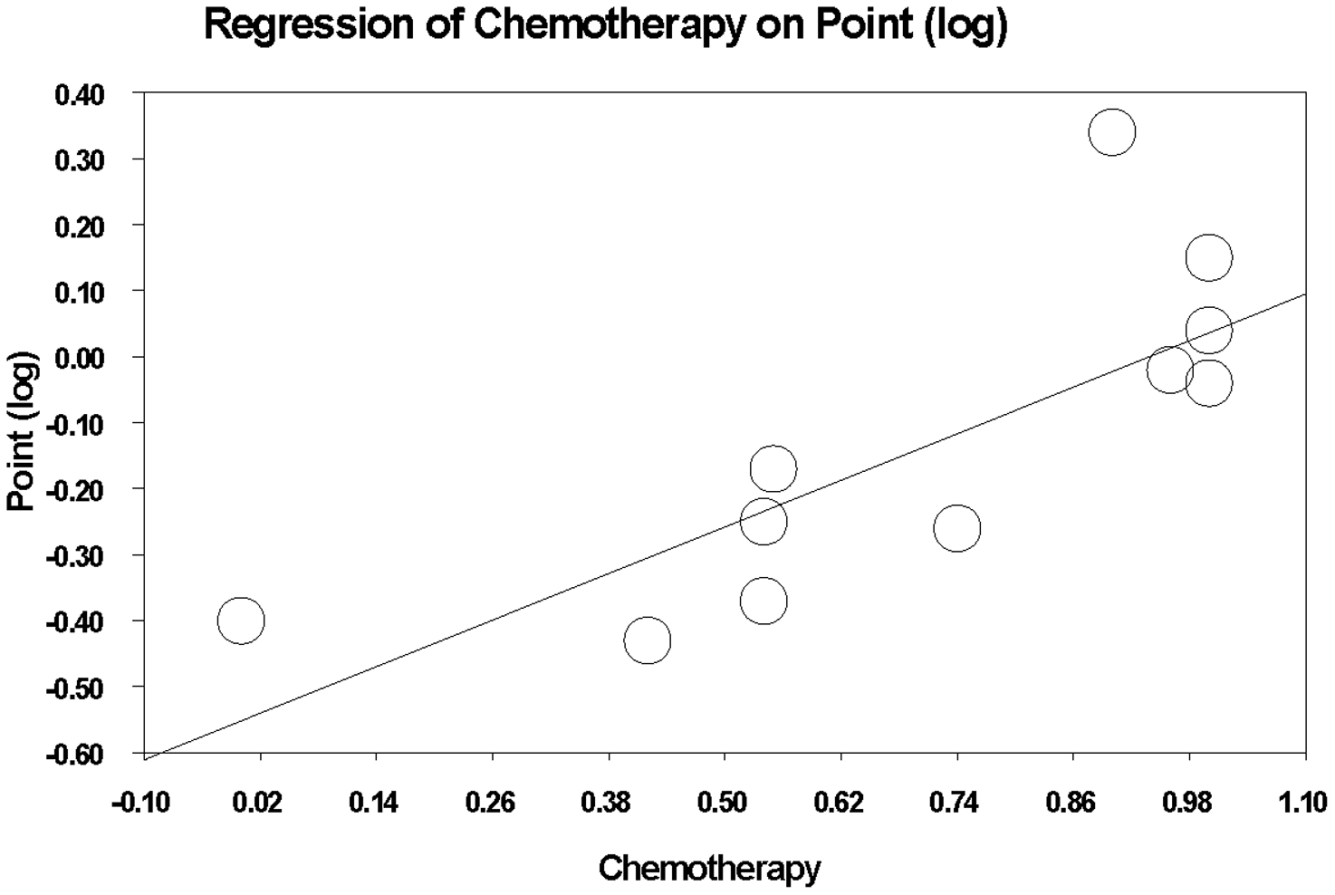
Meta regression of hazard ratios of overall survival in individual studies assessing the effect of percentage of patients receiving chemotherapy in addition to endocrine therapy.

In meta-regression (Figure 4), chemotherapy was negatively associated with HR of overall survival (coefficient, −0.23; standard error, 0.144).

### Adverse Events

The rate of grade 3 or 4 adverse events was reported in thirteen trials [14]–[16], [18], [20], [22], [25]–[26], [28], [56]–[58], including three studies [56]–[58] that evaluated BP for reducing skeletal events without survival assessment, as depicted in Table 2. Bisphosphonate therapy resulted in a lower rate of bone fractures in the intervention arm (RR = 0.59, 95% CI = 0.42 to 0.83). The rate of osteonecrosis of the jaw (ONJ) was higher in the BP arm than in the control arm (RR = 7.53 95% CI = 2.91 to 19.50). To note, that RR was similar by using either the Mantel-Haenszel or Peto method. Pyrexia was also significantly higher in the BP arm (RR = 3.36, 95% CI = 2.61 to 4.32). The incidence of all constitutional grade 3 to 4 adverse events was not statistically significantly affected by BP therapy compared to control as depicted in Table 2.

**Table 2:** Relative risks (RRs) of grade 3–4 adverse events for patients who received BP in addition to standard therapy *vs* those who received standard therapy only.

ONJ	7.53 [2.91, 19.50]
Fractures	0.59 [0.42, 0.83]
Arthralgia	0.98 [0.76, 1.26]
Vomiting	1.14 [0.82, 1.58]
Pyrexia	3.36 [2.61, 4.32]
Hot flushes	0.95 [0.86, 1.05]
Fatigue	0.83 [0.61, 1.11]
Bone pain	1.35 [0.98, 1.87]

## Discussion

The results of our meta-analysis demonstrate a positive effect for BP on survival outcomes when administered in the adjuvant setting, only in post-menopausal patients with breast cancer. Meta-regression appraised the effect of confounders such as chemotherapy on the interaction between BP use and overall survival, and showed a negative association between chemotherapy use and the effect of BP on survival.

The primary endpoint of many BP trials in the setting of early breast cancer was a reduction in skeletal related events, while survival had not been an integral endpoint of the study. We attempted to avoid publication bias by searching for and including in our meta-analysis conference proceedings, databases of ongoing trials, and unpublished data, and indeed in the recent San-Antonio Breast Cancer Symposium (2011) two large scale trials [28]–[29], have been primarily presented (one of which has been fully published later on) and were included in our meta-analysis.

Bisphosphonates have been proven to display high affinity for bone and to inhibit bone resorption by osteoclasts [34]–[35]. Newer generation, nitrogen-containing bisphosphonates, such as alendronate, risedronate, pamidronate, ibandronate, and zoledronic acid are considered an integral part of treatment to reduce the risk for skeletal related events in patients with bone metastases [36]–[39]. Many *in-vitro* studies have portrayed the effect of a variety of BP (both nitrogen containing and non-nitrogen bisphosphonates) to hinder tumor cell adhesion, invasion, proliferation and interplay with the bone microenvironment components such as matrix-metalloproteinases (MMPs) [40]–[41]. Moreover in their well-recognized target, the osteoclast, BP interfere with signaling pathways (e.g. Rac and Ras) required for osteoclast function and survival [42]. Breast cancer cells have a unique predilection to metastasize to bone, whereas the microenvironment is highly receptive to metastatic tumor cells [4].

Two large cohort studies evaluated breast cancer incidence in healthy postmenopausal women treated with bisphosphonates to prevent bone loss and showed a 29–32% statistically significant reduction in the incidence of invasive breast cancer, lending credence for a potential chemo- preventive effect for BP [9]–[10]. The benefit was observed both in ER-positive and ER-negative tumors, implying that the mechanisms may be hormonally-independent and might involve pathways such as angiogenesis inhibition, modulation of the immune system or a direct effect on the bone microenvironment.

The biological rationale for the benefit of BP in post-menopausal patients lies in the natural estrogen deficiency at postmenopause which leads to increased osteoclast activity, resulting in decreased bone mineral density (BMD), yet is still considered hypothetical. Bisphosphonates have been shown to be effective in numerous studies in preventing cancer treatment-associated bone loss in postmenopausal women with early breast cancer [42]–[44] mainly due to their effect on the highly activated osteoclasts. Of note, the increase in bone loss is 5-fold higher following treatment with aromatase inhibitors (AIs) than physiological bone loss observed in postmenopausal women with established osteoporosis in the absence of AI therapy, and further induce bone turnover than documented during tamoxifen use [45]–[49]. A recent study by Cheung indicated that the effect of aromatase inhibitors (exemestane) on bone density may be underestimated and that exemestane increases cortical bone permeability thus predisposing the patient to loss of bone strength and non-vertebral fractures [50]–[51].

Moreover, *in vitro* studies have indicated there may be a synergistic effect between zoledronic acid and letrozole on breast cancer cell lines [52]. This has been the leading rationale for the design of the Z-FAST, ZO-FAST, E-ZO-FAST that evaluated zoledronic acid in combination with AI [18], [20], [26]. Premenopausal patient who become pharmacologically-induced postmenopausal by Gonadotropin Relasing Hormone agonists (GnRHa) may benefit from the addition of bisphosphonates, since bone turnover is further enhanced in premenopausal women who undergo chemical ovarian suppression' as indicated in the ABCSG-12 trial [22]. Meta-regression that assessed the effect of chemotherapy on the association between BP use and survival indicated that the use of chemotherapy may lessen the effect of BP on survival. This result may elucidate the lack of survival benefit in few of the included studies in which chemotherapy was administered in a major portion of the study population [19], [24], [28]–[29]. On the contrary, in the ABSCG-12 which demonstrated an impressive survival benefit for the addition of zoledronic acid, less than 5% of the patients received chemotherapy.

Meta-analysis of the adverse events as reported in the included studies revealed a significant increase in pyrexia as well as osteonecrosis of the jaw (ONJ). Nevertheless, the incidence of ONJ is extremely low (0.24%, according to a recent meta-analysis [53]) and is therefore still considered a non-frequent adverse effect of BP. Although oral bisphosphonates are often associated with gastrointestinal adverse effects while intravenous bisphosphonates have been associated with infusion rate-dependent effect on renal function, the incidence of these adverse events was not statistically significant in all of the studies. Adequate hydration, serum creatinine monitoring and dose reductions in patients with renal impairment prior to BP administration are mandatory in the oncology setting and may support the lack of renal toxicity in the studies.

### Limitations

Several limitations of this analysis must be acknowledged. The study population varies between trials with regard to the risk of recurrence: whereas in few of the large-scale trials most patients had positive lymph nodes at diagnosis [24], [28]–[29], indicating an a-priori worse prognosis than for patients with N0 disease as in the ABCSG-12 trial. Chemotherapy administration differs among studies; in the AZURE trial almost all patients received chemotherapy, in few trials all patients received chemotherapy [16], [19], [29] and in several studies more than half of the patients received chemotherapy [15], [17]–[18], [20], [26], [28]. On the contrary, in the ABSCG-12 less than 5% received chemotherapy, also reflecting the good prognosis of the included population. This heterogeneity prompted us to perform meta-regression which indeed demonstrated that chemotherapy administration correlates with a lack of effect to BP. It should be acknowledged that the result of the meta-regression may reflect more the higher risk group in which chemotherapy was used and less so the effect of chemotherapy itself; along with the known limitations of a meta-regression [31]. Although meta-analysis of the subgroup of post-menopausal patients was feasible due to a separate report on the outcome of this population, in some of the trials the study population was not stratified by menopausal status. Moreover, the trials differ by the specific BP that had been employed. Upon *in vitro* studies, zoledronic acid may exhibit more potent anti-cancer effect than other BP.

The schedule and duration of the intervention varies between studies and could account for the apparent discrepancy in survival. Pre-clinical studies have demonstrated the activation of T cells by bisphosphonate as their key anti-tumor effect which is paradoxically reduced with each subsequent administration of BP. Thus, frequent administration as in few of the trials may result in a reduced anti-tumor effect [54]–[55].

### Implication for research

As indicated by our results, the group of patients that tends to benefit the most from the addition of BP in the adjuvant setting is the post-menopausal population, especially those who are not treated with chemotherapy. In the era of tailored therapy, the use of molecular techniques enables to individualize prognostic and predictive genetic traits by the 21-gene recurrence score (RS) assay (Oncotype Dx) or a microarray of 70-gene expression profile (Mammaprint) identifies subsets of patients with estrogen receptor positive tumors, who are not treated with chemotherapy; it would therefore be of value to elucidate the role of BP in a randomized trial in this population. This design may define clearly the potential benefit of bisphosphonates while the use of chemotherapy would not hamper their potential anti-tumor effect.

### Implications for practice

Our meta-analysis provides a reflection of the new mounting and highly significant data published in the last 3 years which shed new light on the results of a former published meta-analysis on this matter both regarding efficacy and safety of BP in early breast cancer [53]. In view of the lack of evidence for the effectiveness of BP on survival in patients treated with chemotherapy or in premenopausal patients, this treatment should be considered in addition to endocrine treatment only in a subset of postmenopausal patients who are not scheduled for chemotherapy or as part of a clinical trial. Furthermore, the protective effect of BP on bone loss associated with AIs in postmenopausal women with early breast cancer as demonstrated in former studies further rationalize adopting these agents in this population. The addition of BP to the armamentarium of adjuvant treatments for early breast cancer should be further delineated in large scale RCTs which may unravel the specific subgroup that may benefit from this intervention.

## Supporting Information

Checklist S1 **Checklist items according to PRISMA criteria.**
(DOC)

Figure S1 **Sensitivity analysis of survival according to median follow up duration.** Hazard ratios for each trial are represented by the **squares**, the size of the square represents the weight of the trial in the meta-analysis, and the **horizontal line** crossing the square represents the 95% confidence interval (CI). The **diamonds** represent the estimated overall effect based on the meta-analysis random effect of all trials.
(TIFF)

Table S1 **Quality assessment of included studies according to Cochrane criteria.** Quality assessment: Low risk-adequate (central randomization; numbered or coded bottles or containers; drugs prepared by the pharmacy; serially numbered sealed opaque envelopes; other convincing) Unclear – not reported.
(PDF)
