# Supplementary figures and images for: Bisphosphonates in the Adjuvant Setting of Breast Cancer Therapy—Effect on Survival: A Systematic Review and Meta-Analysis
Source: PLoS One. 2013 Aug 26;8(8):e70044. doi: 10.1371/journal.pone.0070044 (PMC3753308; doi:10.1371/journal.pone.0070044)

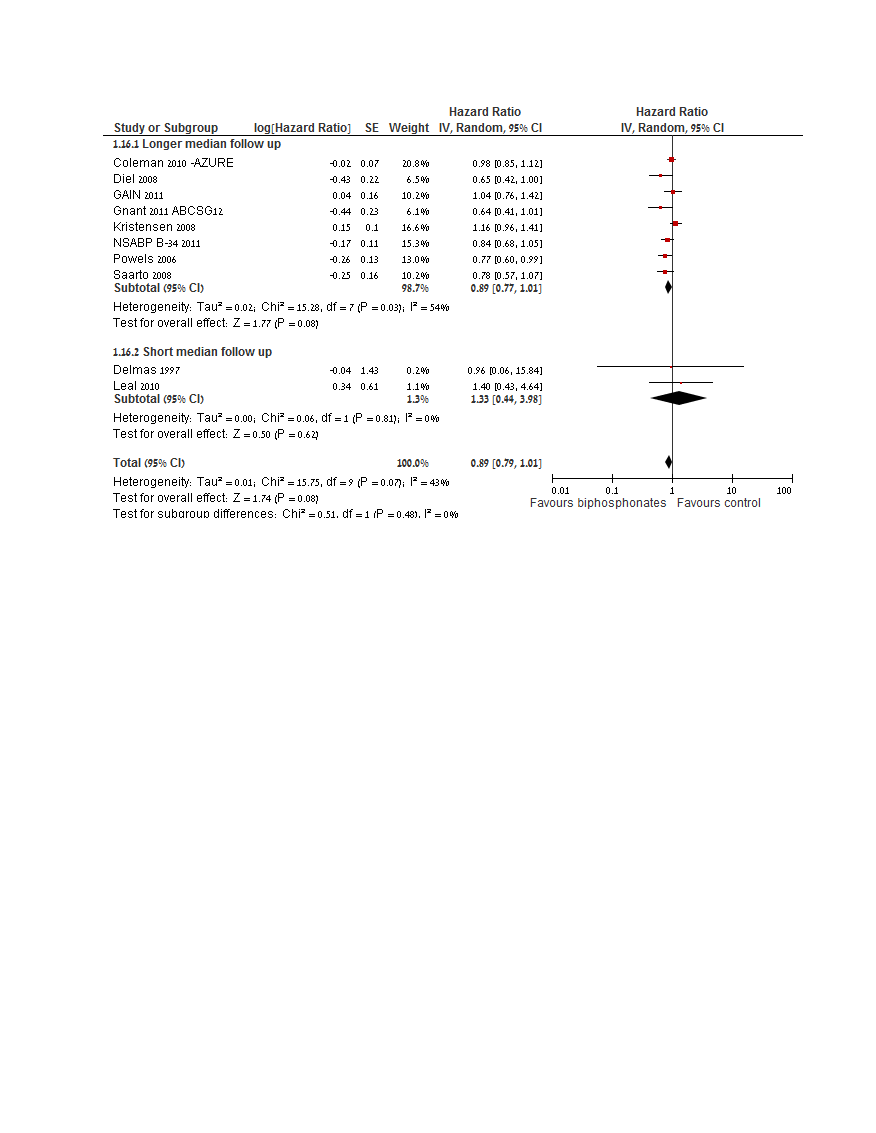

Supplement: Figure S1 — Sensitivity analysis of survival according to median follow up duration. Hazard ratios for each trial are represented by the squares, the size of the square represents the weight of the trial in the meta-analysis, and the horizontal line crossing the square represents the 95% confidence interval (CI). The diamonds represent the estimated overall effect based on the meta-analysis random effect of all trials. (TIFF) [file pone.0070044.s002.tiff]
