# Supplementary material for: Bisphosphonates in the Adjuvant Setting of Breast Cancer Therapy—Effect on Survival: A Systematic Review and Meta-Analysis
Source: PLoS One. 2013 Aug 26;8(8):e70044. doi: 10.1371/journal.pone.0070044 (PMC3753308; doi:10.1371/journal.pone.0070044)
Supplement: Table S1 — Quality assessment of included studies according to Cochrane criteria. Quality assessment: Low risk-adequate (central randomization; numbered or coded bottles or containers; drugs prepared by the pharmacy; serially numbered sealed opaque envelopes; other convincing) Unclear – not reported. (PDF) [file pone.0070044.s003.pdf]

First Author, Year, Publication status	Random sequence generation (selection bias)	Allocation concealment (selection bias)	Blinding of participants and personnel (performance bias)	Incomplete outcome data (attrition bias)	Free of selective reporting (reporting bias)
Delmas, 1997<br>Published PR	Unclear risk	Unclear risk	Low risk	Low risk	Low risk
Saarto,<br>2004<br>Published PR	Unclear risk	Unclear risk	Unclear risk	Low risk	Low risk
Powels, 2006<br>Published PR	Low risk (numerically ordered and coded packages containing either oral clodronate or placebo according)	Low risk (Central)	Low risk	Low risk	Low risk
Diel,<br>2008<br>Published PR	Unclear risk	Unclear risk	Unclear risk	Low risk	Low risk
Kristensen, 2008<br>Published PR	Unclear risk	Unclear risk	Unclear risk	Unclear risk	Unclear risk
Brufsky,<br>Z-FAST, 2011<br>Published PR	Unclear risk	Unclear risk	Unclear risk	Unclear risk	Low risk
Coleman EZO-FAST, 2009<br>Conference proceeding	Unclear risk	Unclear risk	Unclear risk	Unclear risk	Unclear risk
Leal,<br>2010ת<br>Published PR	Unclear risk	Unclear risk	Unclear risk	Unclear risk	Low risk
Eidtmann, 2010,<br>ZO-FAST<br>Published PR<br>+ update from SABCS 2011	Unclear risk	Unclear risk	Unclear risk	Unclear risk	Unclear risk
Gnant, ABCSG12, 2011<br>Published PR<br>+ update from SABCS 2011	Low risk (computer-generated adaptive randomisation method to assign treatment groups)	Low risk (Central)	Low risk	Low risk	Low risk
Coleman, AZURE, 2011<br>Published PR	Unclear risk	Low risk (Central)	Low risk	Unclear risk	Low risk
NSABP B34, 2012	Low risk (coin minimisation approach)	Low risk (Central)	Low risk	Low risk	Low risk
GAIN,<br>2011, Conference proceeding	Unclear risk	Unclear risk	Low risk	Unclear risk	Unclear risk

Quality assessment: Low risk-adequate (central randomization; numbered or coded bottles or containers; drugs prepared by the pharmacy; serially numbered sealed opaque envelopes; other convincing) Unclear – not reported
